# Ozone
*L'Ozone, ou Recherches Chimiques, Météorologiques, Physiologiques et Médicales, sur l'Oxygène Électrisé. Par H. Scoutetten. Paris: Libraire de Victor Masson. 1856.On the Relative Value of the Ozonometers of Drs. Schönbein and Moffat. By T. Herbert Barker, M.D. London: 1856.


**Published:** 1856-10

**Authors:** 


### OZONE.*

SINCE the time when Schönbein first pointed out the existence in the atmosphere of a peculiar principle, to which he gave the name of ozone, and which he supposed to be identical with the peculiarly smelling product developed in the electric discharge, great attention has been paid to the matter, both in this country and on the continent of Europe.

We have now before us, in a volume published within the last few days, the largest and latest work on this interesting subject. M. Scoutetten has, indeed, undertaken here to represent all that has been said regarding ozone, its nature, its development, and its influence. That our author has failed in writing a fair and extended review, is a fact that must be acknowledged, when it is stated that he has not referred to one English authority. Glaisher, Moffat, Barker, and many others, including even Professor Faraday, have, with their

---

* L'Ozone, ou Recherches Chimiques, Météorologiques, Physiologiques et Médicales, sur l'Oxygène Électrisé. Par H. SCOUTETTEN. Paris : Libraire de Victor Masson. 1856.

On the Relative Value of the Ozonometers of Drs. Schönbein and Moffat. By T. HERBERT BARKER, M.D. London : 1856.

works, been entirely ignored or overlooked by M. Scoutetten. The views propounded in this new treatise run briefly as follows.

Ozone is oxygen positively electrified; it is produced naturally in the atmosphere everywhere; it is formed during the natural electrical discharges, and during certain chemical processes; it is the most powerful oxidising agent known; its presence is more strongly marked in the night than in the day; and it is irregular in its progress. From observations carried on at Metz, during six months, M. Scoutetten found that in a close, narrow street, with a cesspool at its head, the presence of ozone was only seen to be developed four times, though looked for daily, night and morning. During the same period, observations were taken at a window of his own apartment, looking southward, and above a garden; as well, also, at the military hospital, an isolated building. In both these places the indications of ozone were always given, being sometimes very strongly marked. He infers, therefore, that the ammoniacal and other emanations of close localities check greatly the production of ozone; and, although experiment proves that ozone is more freely indicated at great heights, as on a mountain, yet in reality it does not progressively increase in quantity according to elevation, but diminishes in the lower atmospheric strata from local influences.

A curious fact in the development of ozone is, that it is produced with peculiar freedom over a surface of earth covered with vegetation, and over water. A series of experiments are recorded, also, to show that there is no development of ozone in an inhabited room. It would seem, indeed, that the presence of one individual is sufficient to prevent the detection of ozone; for Scoutetten found that in his own bedroom, which was perfectly lightsome, and well ventilated, the ozone paper remained perfectly white, although, when exposed to the free air on the outside of one of the windows, it was always more or less tinted. In the hospital of Versailles, Dr. Berigny found the same result. The non-colouration of the ozone test-paper in the inhabited room is, as we opine, due possibly to the presence of ammonia, which, according to our researches, is always being evolved from the animal body.

Some remarks and experiments on the physiological effects of ozone tend to show, that in a large dose ozone rapidly produces excitement of the respiration, spasms of the bronchial tubes or vessels of the thorax, and, more slowly, upon its action being prolonged, coryza, intense bronchitis and pneumonia. In proper proportions in the air it is, however, indispensable to the due accomplishment of the living functions. In confined places, where its presence is not, plants and men alike become blanched; the skin in the man becomes pallid, the blood loses colour, the lymph predominates, all the tissues soften, and serious diseases (of the adynamic type) break out.

The last sections of the book are devoted to an inquiry into the connexion supposed to exist between the presence or absence of ozone, and the existence or prevalence of certain diseases. The opinions here recorded from various observers are contradictory. M. Schifferdecker is quoted as expressing the view that there is no connexion between the presence of ozone and diseases of the chest. His view is opposed by Scoutetten, who traces in the records of the military hospitals of Metz an exact correspondence between the variations of ozone and the number of bronchial affections. It was not, however, on the same days when the ozone papers were tinted deeply that the patients entered the hospital, but only on the following day, or many days afterwards. This occurrence is explained away by the hypothesis of a period of incubation; rather a shaky argument.

Some further observations supporting the hypothesis of a connexion between ozone and some chest affections are supplied from the labours of M. Böckel, but these seem to us to be built on insufficient data.

The work of M. Scoutetten concludes with a short chapter on the employment of ozone in therapeutics. The book is well worthy the attention of all our readers, and we regret much that, from the fact of its very recent publication, we are not able to give a more complete analysis of its contents.

Dr. Barker's *brochure* is written simply to show the superiority of Dr. Moffat's ozone papers over those of Professor Schönbein, a superiority which seems to be established beyond all doubt. The comparison of the two papers was carefully made, and extended over a period of eighteen months, daily. During this period there were 122 days on which Schönbein's ozonometer indicated the presence of ozone, while Moffat's gave the indication on 207 days, and never failed to receive a tinge whenever Schönbein's gave the indication. The total amount of ozone indicated by Schönbein's paper, during the entire period of observation, is represented by the number 353, while Moffat's paper registered 793. The mean monthly amount by Schönbein's ozonometer was 19·50; by Moffat's, 44·05. The mean daily amount by Schönbein's was 1·70; by Moffat's, 3·83.

